# The Premasseteric Branch of the Facial Artery: A Review and Translation of Adachi’s Work

**DOI:** 10.7759/cureus.10538

**Published:** 2020-09-18

**Authors:** Stephen J Bordes, Sina Zarrintan, Joe Iwanaga, Marios Loukas, R. Shane Tubbs

**Affiliations:** 1 Anatomy, St. George's University, St. George's, GRD; 2 Cardiac/Thoracic/Vascular Surgery, Tabriz University of Medical Sciences, Tabriz, IRN; 3 Neurosurgery, Tulane Center for Clinical Neurosciences, Tulane University School of Medicine, New Orleans, USA

**Keywords:** premasseteric branch, facial artery, anatomist, anatomy, maxillofacial surgery

## Abstract

The premasseteric branch of the facial artery is a variable posterior branch that is closely associated with the anterior border of the masseter muscle. Since its first description, the premasseteric branch has been described using different terms such as the masseteric or posterior branch of the facial artery. While the artery’s anatomy is known, it is infrequently discussed in the literature. This manuscript reviews the artery’s origin, course, and importance during maxillofacial procedures, especially those involving manipulation of the masseter. We also provide a translation of Adachi’s 1928 German text describing the branch.

## Introduction and background

Buntaro Adachi (1865-1945) was a Japanese physician, anatomist, and anthropologist well-known for his research and depictions of anatomical variation in man [1,2]. Adachi was born and raised in Honshu, Japan; he attended Tokyo Imperial University and later taught at the University of Okayama Medical School [1]. Adachi’s studies in human anatomy then brought him to Strasbourg, Germany from 1899-1904, after which he returned to Japan as a professor at Kyoto Imperial University [1]. Following mandatory retirement from the university in 1925, Adachi became the president of Osaka Medical College [1]. His two most famous works, Das Arteriensystem der Japaner (1928) and the two-part Das Venensystem der Japaner (1933 and 1940), originally written in German, are still widely recognized and highly regarded in human vascular and variation research [1-5]. In his 1928 publication, Adachi extensively described a variation of the facial artery, which he named the “ramus premassetericus,” also known as the premasseteric or posterior branch of the facial artery [5]. Anatomical knowledge of the artery is important during craniofacial procedures involving the masseter muscle; however, modern texts have yet to standardize its terminology, which has led to some ambiguity in the literature regarding its origin, course, and variation [6]. Herein, we provide a translation of Adachi’s description of the “ramus premassetericus” as well as a review of the literature regarding this arterial branch to summarize existing knowledge so that we can apply it toward better and safer surgery.

## Review

Translation of Adachi’s “ramus premassetericus” of the maxillary artery

The maxillary artery and the anterior facial vein are close to each other at the jaw edge, whereas, in the face, they rise divergently so that the former runs in front of the latter (the maxillary artery is accompanied by particularly fine double veins, as previously mentioned by Bardelebens and Sobotta). From there, when the artery and vein separate from each other, in the majority of faces, the vein accompanies a fine arterial branch emerging acute-angled from the maxillary artery on the edge of the mandible. The branch, which is called the "ramus premassetericus,” thus turns at the front edge of the masseter muscle upward, but ends very soon or anastomoses with the surrounding arteries.

**Figure 1:**
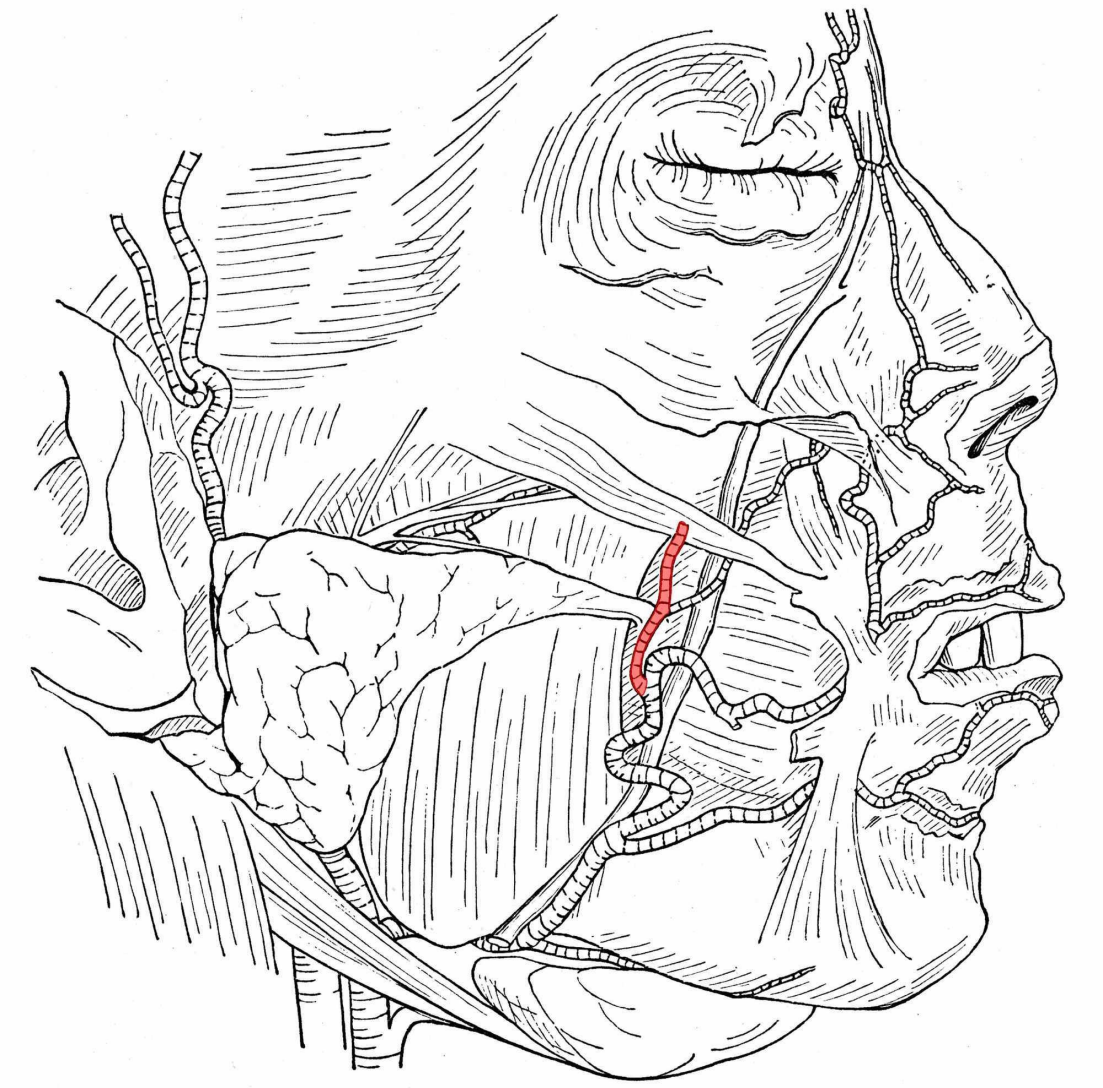
A strongly developed ramus premassetericus that accompanies the anterior facial vein in a 23-year-old male Adapted from Adachi B's Anatomie der Japaner 1: Das Arteriensystem der Japaner. Kyoto; 1928, with modification [5]

The ramus premassetericus rarely forms a considerable artery (Figure 1), and almost only when the continuation of the maxillary artery, which runs in front of the ramus, is weak.

Among 132 halves of the face (66 cadavers: 52 male, 14 female; 1907 and 1908), the said ramus premassetericus was four times (male: two times right; female: two times left) very strong, about as strong as the usual continuation of the maxillary artery running in front of the ramus or even stronger. So in this case, in the face, the maxillary artery, which accompanies the anterior facial vein, turns into this ramus while the actual continuation of the maxillary artery is a branch of the ramus premassetericus or is simply missing.

**Figure 2:**
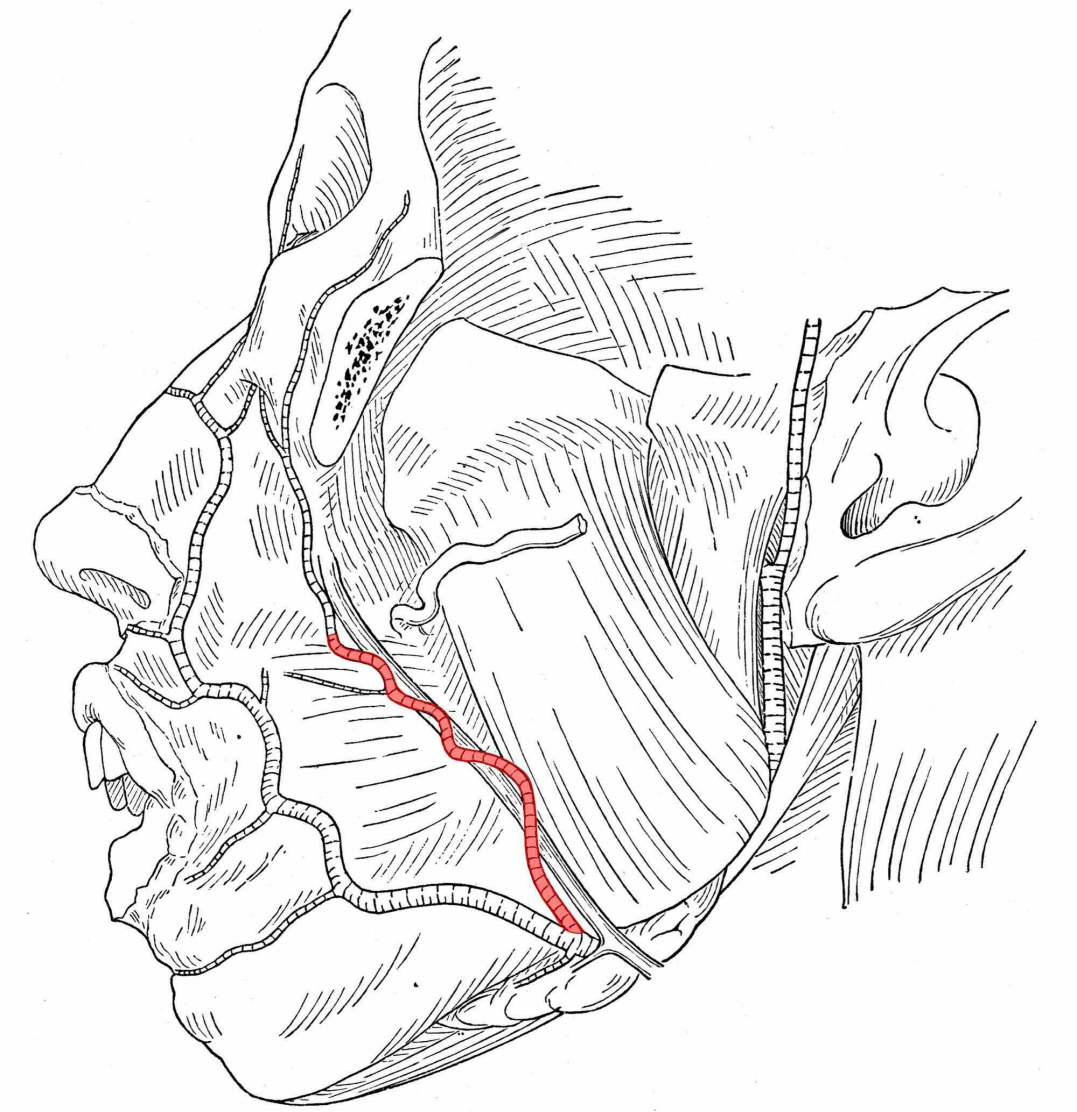
The ramus premassetericus is strongly developed in the lower half of the face and the actual maxillary artery is missing in a 19-year-old male Adapted from Adachi B's Anatomie der Japaner 1: Das Arteriensystem der Japaner. Kyoto; 1928, with modification [5]

In Figure 2, we present a somewhat special case (the case was encountered by chance in 1918) where the ramus premassetericus is strongly developed in the lower half of the face and the actual maxillary artery is missing.

The latter is present, however, in the upper half of the face and yet stronger than the continuation of the ramus premassetericus.

Even with stronger development, the ramus premassetericus offers no striking image when the anterior facial vein is disregarded. At the preparations where the vein already has been removed or displaced from its natural position, it is often doubtful whether it is an ordinary maxillary artery or a very developed ramus premassetericus. Such preparations are not included in the aforementioned 132 halves of the face.

In several textbooks (Cunningham, Murrich in Piersol, Poirier, Testut), the ramus is mentioned as the “masseteric branch,” “branches massétérines,” or “massétérine inferieure.” By name, the description of Murrich is applicable. Various pictures of the ramus are also found in the atlases of Broesike (Vol. II, figs. 350-354) and Toldt (Gefäßlehre, figs. 968, 969, and 1046). Broesike called the branch “A. premasseterica.” In a specimen of veins of Toldt (fig. 1046), the anterior facial vein accompanies a strong arterial branch.

Literature review

Branches of the Facial Artery

The facial artery provides the blood supply for a significant portion of the face. The artery originates from the external carotid artery (ECA) and gives off cervical (ascending palatine artery, tonsillar branch, submental artery, and glandular branches) and facial branches (superior and inferior labial branches, lateral nasal branch, and angular artery) anteriorly [6-10]. Variations exist in which the facial artery produces posterior branches, but these branches often remain unnamed [8].

Anatomy of the Premasseteric Branch of the Facial Artery

**Figure 3:**
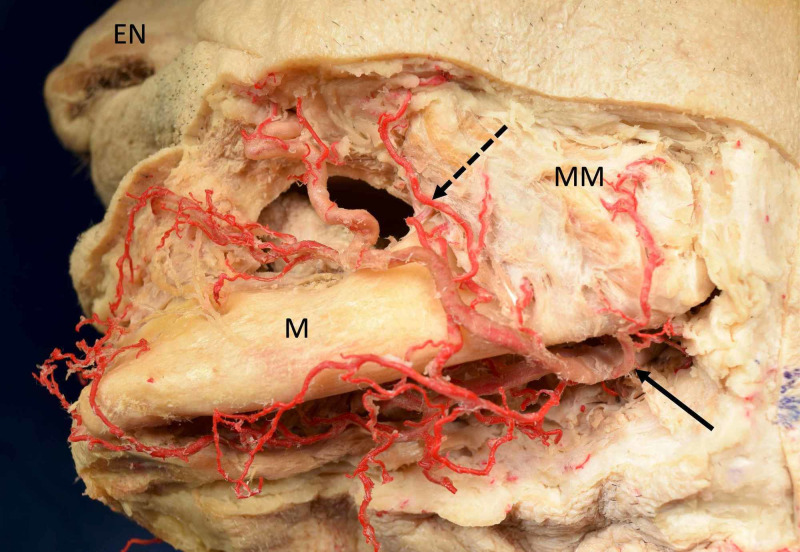
Left premasseteric branch (dotted arrow) of the facial artery (solid arrow) in a Caucasian cadaveric specimen EN: external nose; M: mandible; MM: masseter muscle

The premasseteric, also known as masseteric or posterior, branch of the facial artery is a better known posterior branch that was named as early 1928 by Adachi; however, it has seldom been discussed in the literature (Figure 3) [5].

The premasseteric branch of the facial artery originates in the submandibular region and crosses the ramus of the mandible and travels near the facial vein along the anterior border of the masseter to supply surrounding tissues [5,6,8,11-13]. It is found to divide into superficial and deep branches that usually pierce the masseter and terminate in the region of the parotid duct [6,8,12]. The artery has been seen to anastomose with the superior masseteric branch of the transverse facial artery as well as the middle and inferior masseteric branches of the maxillary, facial, or ECAs [5,6,11]. The arterial branch is generally small, but variations do exist in which the vessel is as large as the facial artery itself [5,6]. According to Mağden et al. (2009), the mean diameter of the premasseteric branch at its origin was 1.12 mm (range: 0.60-2.10 mm) [6]. 

Blood Supply of the Masseter Muscle and Surgical Considerations

The artery has been implicated as a potential source of complication in craniofacial procedures specifically involving the masseter muscle, which is supplied by masseteric branches of the facial, transverse facial, and maxillary arteries [6,7,14]. Regarding the blood supply of the masseter, Hwang et al. (2001) proposed using the terms superficial and deep middle masseteric arteries originating from either the ECA or common carotid artery [15]. A study by Ariji et al. (2001), using Doppler sonography, investigated the detection rate of the arteries that supply the masseter and revealed that the masseter branch of the facial artery was detected on 100% of sides (72 sides) [14]. According to Won et al. (2012), the masseteric branch of the facial artery and masseteric branch of the premasseteric artery were observed in 88% (22/25) and 56% (14/25) of specimens, respectively [16].

Typically, this premasseteric branch exists as a single vessel; however, reports have noted the facial artery giving rise to multiple premasseteric branches [7,11]. As a result, knowledge of potential variation is crucial in order to reduce the risk of transection during maxillofacial and plastic surgery procedures such as musculo-mucosal flaps, treatment of facial palsy, benign masseteric hypertrophy, parotid tumor resection, and lower lip repair, to name a few [6,8,11,17,18]. Even for general dentists and oral surgeons, the premasseteric branch might cause bleeding as the facial artery in this area travels near the buccal periosteum in the lower molar region and can be injured during oral surgery [19].

Terminology

This literature review has revealed that there are several different terms for the premasseteric branch of the facial artery. Moreover, some articles have used similar terms for different structures and vice versa (Table 1). Therefore, this needs to be amended for future studies and for the readers’ better understanding.

**Table 1:** Overlapping terminology concerning blood supply to the masseter

Artery	Terminology used	Author
Premasseteric branch of the facial artery	Ramus premassetericus	Adachi (1928) [3]
	Premasseteric branch of the facial artery	Mağden et al. (2009) [6]
		Nayak (2019) [7]
	Posterior (premasseteric) branch of the facial artery	Padur et al. (2019) [8]
	Masseteric branch of the facial artery	Arjii et al. (2001) [14]
	Masseteric artery	Marinho et al. (1991) [11]
	Premasseteric artery	Vasudha et al. (2018) [12]
		Won et al. (2012) [16]
Posterior branch of the premasseteric artery that supplies the masseter	Masseteric branch of the premasseteric artery	Won et al. (2012) [16]
Posterior branch of the facial artery inferior to the origin of the premasseteric branch	Masseteric branch of the facial artery	Won et al. (2012) [16]
Masseteric branch of the maxillary artery	Masseteric artery	Hwang et al. (2001) [17]

## Conclusions

Since its detailed description in 1928, the premasseteric branch of the facial artery has been a source of little discussion. Knowledge of the artery and its variations is crucial during maxillofacial procedures in order to avoid complications. While the artery is often small, a large branch or multiple branches can result in significant hemorrhage if the anatomy of the masseter muscle and its surrounding structures is not fully appreciated. This literature review has raised some new questions, i.e., regarding clinical consequences and terminology, to be addressed. Further studies to identify this branch are definitely needed.
